# Improved Fully Convolutional Siamese Networks for Visual Object Tracking Based on Response Behaviour Analysis

**DOI:** 10.3390/s22176550

**Published:** 2022-08-30

**Authors:** Xianyun Huang, Songxiao Cao, Chenguang Dong, Tao Song, Zhipeng Xu

**Affiliations:** 1 Scientific Research Post, Suzhou Institute of Metrology, Suzhou 215128, China; 2 College of Metrology and Measurement Engineering, China Jiliang University, Hangzhou 310018, China

**Keywords:** visual tracking, Siamese tracker, tracking drift, background clutter

## Abstract

Siamese networks have recently attracted significant attention in the visual tracking community due to their balanced accuracy and speed. However, as a result of the non-update of the appearance model and the changing appearance of the target, the problem of tracking drift is a regular occurrence, particularly in background clutter scenarios. As a means of addressing this problem, this paper proposes an improved fully convolutional Siamese tracker that is based on response behaviour analysis (SiamFC-RBA). Firstly, the response map of the SiamFC is normalised to an 8-bit grey image, and the isohypse contours that represent the candidate target region are generated through thresholding. Secondly, the dynamic behaviour of the contours is analysed in order to check if there are distractors approaching the tracked target. Finally, a peak switching strategy is used as a means of determining the real tracking position of all candidates. Extensive experiments conducted on visual tracking benchmarks, including OTB100, GOT-10k and LaSOT, demonstrated that the proposed tracker outperformed the compared trackers such as DaSiamRPN, SiamRPN, SiamFC, CSK, CFNet and Staple and achieved state-of-the-art performance. In addition, the response behaviour analysis module was embedded into DiMP, with the experimental results showing the performance of the tracker to be improved through the use of the proposed architecture.

## 1. Introduction

Visual object tracking has become increasingly important in many application fields, including surveillance, robotics and human–computer interfaces. However, the challenges of reliable tracking due to cluttered backgrounds, occlusion and different illuminations still remain.

Inspired by artificial neural networks [1,2,3,4,5] and deep learning [6], breakthroughs in many areas such as deep learning-based methods have attracted growing interest in the visual object tracking field. According to the network architecture, there are four categories of deep learning trackers: convolutional neural network- or CNN-based trackers, recurrent neural network- or RNN-based trackers, generative adversarial network- or GAN-based trackers and Siamese neural network- or SNN-based trackers [6].

(1) CNN was the first deep learning model to be used in the visual object tracking field due to its powerful representation of a target. Wang [7] proposed a tracking algorithm that used fully convolutional networks pre-trained on image classification tasks, and this performed better than the majority of other trackers regarding both precision and success rate at that time. Nam [8] pre-trained a CNN using a large set of videos with tracking ground truths for obtaining a generic target representation. CNN-based trackers have inherent limitations, including computational complexities and the requirement of large-scale supervised training data.
(2) RNN-based trackers are excellent for dealing with temporal information of video frames, including object movement or motion. Yang [9] embedded a long short-term memory (LSTM) network into a recurrent filter learning network as a means of achieving state-of-the-art tracking. Ma [10] exploited a pyramid multi-directional recurrent network to memorise target appearance. However, RNN-based trackers are generally difficult to train and have a considerable number of parameters that require tuning, and the number of these trackers is limited.
(3) GAN-based trackers can generate desired training positive images in the feature space for tackling the issue of sample imbalance [11]. Guo [12] proposed a task-guided generative adversarial network (TGGAN) to learn the general appearance distribution that a target may undergo through a sequence. As RNN trackers, it is also difficult to train and evaluate GAN-based trackers, so their number is also limited.
(4) Recently, Siamese networks (SNN), which follow a tracking using a similarity comparison strategy, have received significant attention from the visual tracking community due to their favourable performance [13,14,15,16,17]. SNN-based trackers formulate the visual object tracking problem by learning a general similarity map through cross-correlation between the feature representations learned for the target template and the search region. Due to the satisfactory balance between performance and efficiency, SNN-based trackers have become the most widely used and researched trackers in recent years.

Although these tracking approaches can obtain balanced accuracy and speed, some problems must be urgently addressed, the most important of which is the object locating strategy or motion model. With traditional Siamese trackers, the new position of the target is always the location with the highest score in the response map for every input image frame. This strategy can potentially result in tracking drift if distractors exist near to the real target, particularly if one of them has a higher response score than the real target. In order to address this problem, an improved SiamFC tracker based on response map analysis is proposed. Extensive experiments on visual tracking benchmarks including OTB100, GOT-10k and LaSOT demonstrated that the proposed tracker improves the performance in terms of both tracking accuracy and robustness.

The main contributions of this work are as follows:

- A new distractor detecting method is proposed that analyses the response map without training. Following an experimental comparison, it is proven that the proposed response behaviour analysis module can be embedded into other response map- or score map-based trackers as a means of improving tracking performance, making this a common strategy for many other trackers.
- The behaviour of real targets and distractors can be observed and recognised through the analysis of the dynamic pattern of the contours in the response map. This method enables a simple, effective and dynamic analysis of the movement trend of the target and the surrounding distractors over a period of time to be performed for the prediction of the potential impact the distractors have on the target object.
- The performance of the classic SiamFC can be significantly improved through the adoption of the response analysis model during the tracking process. This shows that for certain problems with classical visual target tracking algorithms such as SiamFC, tracking performance can be improved more substantially through the use of well-designed but simple strategies, which do not necessarily require the reconstruction of complex network structures or long training periods.

This paper is organised in the following way. A basic introduction and work relating to Siamese trackers are introduced in Section 2. Section 3 outlines the proposed response analysis method that includes the response map contour, distractor approaching analysis and peak switching strategy. In Section 4, the proposed method is compared to DaSiamRPN [15], SiamFC [17], SiamRPN [18], CFNet [19], CSK [20] and Staple [21] using the OTB100, GOT-10k and LaSOT benchmarks. In addition, the experimental results and analyses are also provided. Finally, Section 5 presents conclusions and suggests future research directions.

## 2. Related Work

The Siamese network consists of two subnetworks with identical network architectures and shared weights. It was initially proposed by Bromley et al. [22] for signature verification, and the pioneering work of the use of the Siamese network in the visual object tracking field is SINT [23], which simply searches for the candidate that is most similar to the exemplar that is provided in the starting frame.

### 2.1. SNN-Based Trackers

Bertinetto et al. proposed a fully convolutional Siamese network (SiamFC) [17] for the estimation of the feature similarity between two frames. SiamFC adopts the Siamese network as a feature extractor, introducing the correlation layer for combining response maps, and the position of the target is determined by locating the maximum value of the response map.

Following the proposal of the classic SiamFC, many further works have been proposed on its basis, including CFNet, DCFNet, RASNet, SiamRPN, CHASE and COMET. CFNet [19] interprets the correlation filters as a differentiable layer in a Siamese tracking framework, thereby achieving end-to-end representation learning. However, the performance improvement is limited in comparison to SiamFC. In order to improve the tracking performance when faced with challenges such as partial occlusion and deformation, FlowTrack [24] exploits motion information in the Siamese architecture as a means of improving the feature representation and tracking accuracy. RASNet [25] was proposed by Wang et al. and embedded diverse attention mechanisms into the Siamese network for adapting the tracking model to the current target. For more accurately estimating the target bounding boxes, Li et al. integrated the regional proposal network (RPN) into the Siamese network and proposed the SiamRPN tracker [18]. The results demonstrated superior tracking performance in comparison to classical trackers with the presence of RPN. Following the proposal of the SiamRPN tracker, many researchers have attempted to improve tracker performance. One typical tracker is DaSiamRPN [15], which utilises a distractor-aware module for performing incremental learning of background distractors. SiamRPN++ [13] made further improvements based on DaSiamRPN, using a spatial-aware sampling strategy and training a ResNet-driven Siamese tracker with a significant performance gain. CHASE [26] was proposed by Marvasti-Zadeh et al., and it is a novel cell-level differentiable architecture search mechanism with early stopping for automating the network design of the tracking module. It has the objective of adapting backbone features to the objective of Siamese tracking networks during offline training. In order to address the problem of tracking an unknown small target from aerial videos at medium to high altitudes, the researchers also proposed a context-aware IoU-guided tracker (COMET) [27] to exploit a multitask two-stream network and an offline reference proposal generation strategy. Several trackers have recently been introduced using transformers, including TransT [28] and ToMP [29]. They have gained significant attention in the visual tracking community. Similar to Siamese-based trackers, these transformer trackers take a pair of image patches as the inputs of the backbone network and employ a feature fusion network consisting of multiple self- and cross-attention modules.

### 2.2. Discriminative Object Representation and Improvement Solutions

The object representation model plays a crucial role in all visual tracking algorithms. A good representation model can help a tracker distinguish between real targets and distractors. A disadvantage of Siamese trackers is poor performance when distractors are close to the true target, as the Siamese network does not have the strategy of discovering distractors during tracking and is only concerned with the highest score of the response map in tracking without any focus on the background clutter situation. Many solutions have been proposed by scholars for solving this problem. They can be classified into the following five categories: (1) Learning distractor-aware. Zhu et al. [15] discovered that the imbalanced distribution of training data makes the learned features less discriminative, proposing the DaSiamRPN algorithm. This method introduced a new sampling strategy and made the model focus on semantic distractors. Similarly, target-aware deep tracking (TADT) [30] chose the target-aware features based on activations to represent the targets. As both trackers utilised pre-trained deep features, and due to the fact that the targets of interest can be arbitrary objects in visual tracking, the problem of being less effective in modelling arbitrary targets to distinguish them from the background still exists.

(2) Combing confidence map. R-FCSN [31] adaptively weighted each region response as a means of forming a joint confidence map. This confidence map placed greater emphasis on reliable regions and eliminated the clutter that is caused by drifting regions. LTSN [32] used a multi-confidence map strategy as a means of improving the adaptiveness of appearance changes and background distractors. The advantage of these algorithms is that they require no training and are fast, but the disadvantage is that they are too simple and do not consider the motion information of the target.

(3) Mining hard samples. Siam R-CNN [33] proposed an embedding network for extracting an embedding vector for every ground truth bounding box that represents the appearance of the object. In this way, the tracker discovered hard examples for re-detection conditioned on the reference object through the retrieval of objects from other videos. DaSiamRPN also used hard sample mining technology to improve object representation. Mining and training hard samples represent an incredibly useful method that leads to the improvement of the performance of distinguishing similar objects, but finding and training hard samples are generally quite difficult.

(4) Integrating background appearance. DiMP [34] proposed an end-to-end architecture based on a target model prediction network, which is derived from a discriminative learning loss, and integrated background appearance as a means of achieving state-of-the-art performance.

(5) Using classification components. ATOM [35] designed special dedicated target estimation and classification components, combining them to create a novel tracking architecture. Both DiMP and ATOM utilised a similar state update strategy based on the comparison of the two maximum peaks of the response map. With this strategy, when some distractors were near to the target, the response map scores were below a certain threshold, resulting in the tracking state being labelled as ‘uncertain’. The position had the highest score returned as the new tracking position, which was not reasonable as this could result in tracking drift as the position with the highest score has a greater probability of being a distractor.

Different to the aforementioned Siamese-based trackers where the problems of background clutter and distractors were addressed through training with different network structures or different data samples, this paper proposes a distractor analysis method for tracking without retraining the network based on a Siamese tracker. The proposed method can handle the tracking drift problem, particularly in background clutter scenarios.

**Figure 1:**
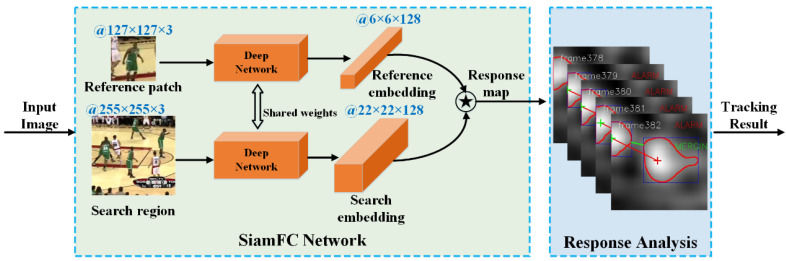
Overview of the proposed tracking method.

The proposed method process is as follows. Firstly, the response map of the SiamFC is normalised to an 8-bit grey image, and the isohypse contours that represent the candidate target region are generated through thresholding. Secondly, the dynamic behaviour of the contours is analysed to ascertain whether there are distractors approaching the tracked target. Finally, a peak switching strategy is used for determining the real tracking position of all the candidates. In addition, a new Siamese network does not need to be constructed for this method, and there is only a need to modify the tracking update process. Following the use of this response analysis method, classic SiamFC tracking performance can be improved to state-of-the-art level. An overview of this proposed method can be seen in Figure 1.

## 3. Proposed Method

In Figure 1, an overview of the visual object tracking method proposed in this paper is presented. In this section, the details of the algorithm will be introduced, including a brief introduction to Siamese trackers, details of response behaviour analysis and the pseudo-code for the proposed method.

### 3.1. Siamese Trackers

With a typical Siamese network, a pair of images $\left(x,z\right)$, where x and z are the target template patch and search patch, is used for training. The images are sent into a deep network as a means of obtaining two feature maps:

(1) $$g_{\rho }\left(p_{t},p_{s}\right)=f_{\rho }\left(p_{t}\right)*f_{\rho }\left(z\right)+b$$

where $f_{\rho }\left(p_{t}\right)$ is a deep convolution network, ρ is a learnable parameter, b is a scalar offset value, * denotes the cross-correlation operation and $g_{\rho }\left(p_{t},p_{s}\right)$ represents the response map, denoting the similarity between $p_{t}$ and $p_{s}$. The training goal is to enable the maximum value of the response map to correspond to the target position.

During the testing stage, similarities between the target template patch and the search patch are presented by a single channel response map, and the estimated location of the target can be predicted as follows:

(2) $$q=\arg\max f\left(p_{t}\right)*f\left(p_{s}\right)$$

where *q* is the central position of the target.

A more detailed explanation of Siamese trackers can be found in [17].

### 3.2. Improved SiamFC Tracker Based on Response Behaviour Analysis

**Figure 2:**
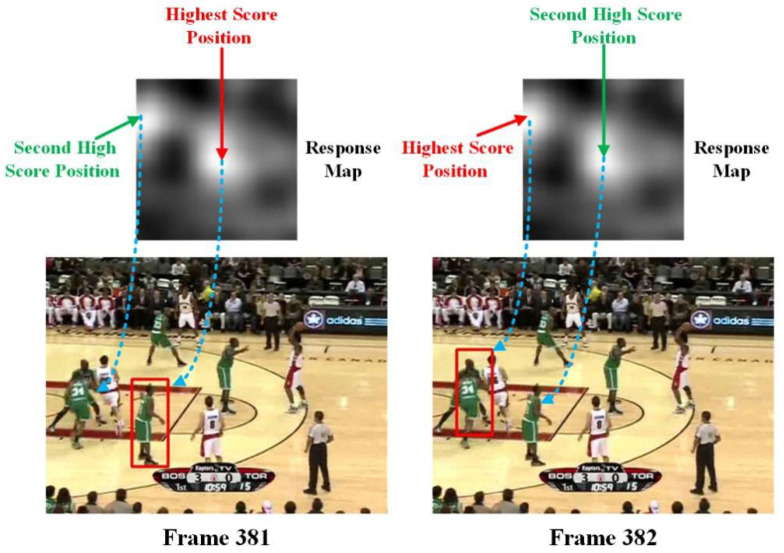
Tracking drift from frame 381 to frame 382.

With traditional Siamese trackers, the new position of the target is predicted using the location with the highest score of the response map for every input image frame. For the frame without background cluttering, the response map is a single model, but if some distractors exist that are similar to the template patch in the searching region, the response map has a general tendency to be multi-model. In certain cases, the distractor has a higher score than the true object. If the tracking strategy involves always changing to the position with the highest score in every frame, the tracking will drift to other background distractors, as can be seen in Figure 2.

**Figure 3:**
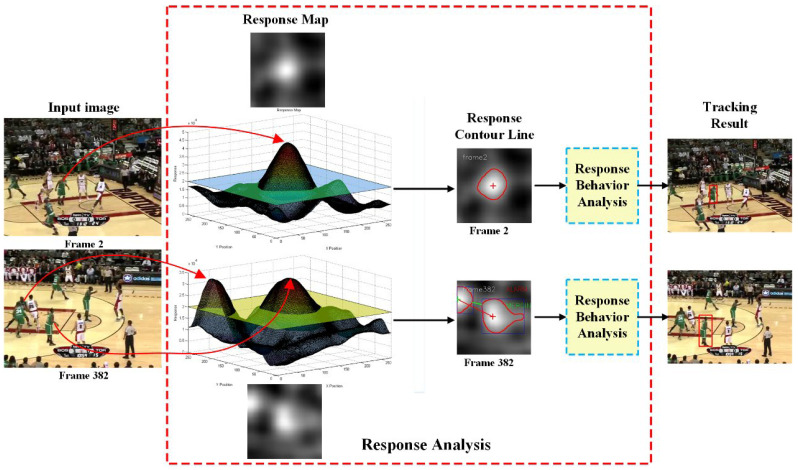
Overview of the proposed response analysis.

Detailed analysis of the response map is essential for improving tracking performance and addressing this problem. It was found that changing the response map from frame to frame exhibited interesting behaviour that could be used to analyse whether distractors are approaching. Based on this motivation, this paper proposes an improved SiamFC tracker based on response behaviour analysis. An overview of the proposed method can be seen in Figure 3.

#### 3.2.1. Response Isohypse Contour

**Figure 4:**
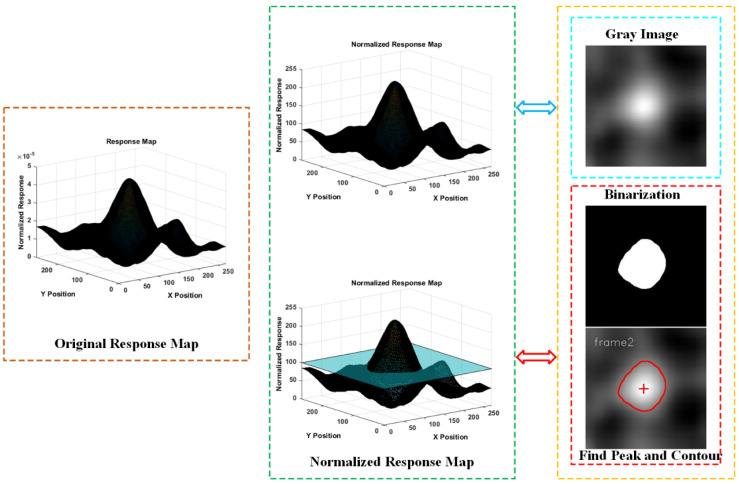
Illustrations of response isohypse contour.

A response map can be normalised to an 8-bit grey-level image where a higher value represents a higher score of the original response map. In this normalised response map, a binarisation operation with a certain threshold is equivalent to drawing a contour plane of the original response map. The blob regions of the binarised image can then be used for analysing the behaviour of the response map frame by frame. An overview of the response isohypse contour method can be seen in Figure 4.

#### 3.2.2. Distractor Approaching Analysis

**Figure 5:**
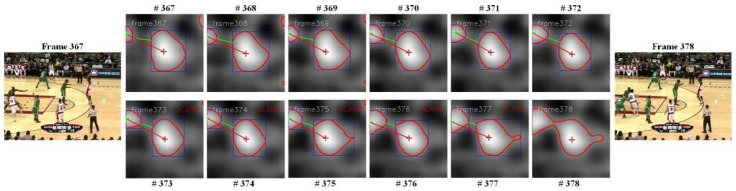
The process of distractor approaching analysis.

An obvious phenomenon can be witnessed when an analysis of the response map is performed. When there is no distractor with a similar appearance around the target, the response map is unimodal, but when an obvious distractor is nearby, it will generally be multi-modal. By transferring the response map to the response isohypse contour map, the situation where there is only one contour in the middle of the map represents tracking without background cluttering, and when the map has more than one contour, this indicates the presence of distractors around the target. In addition, if one contour gradually becomes closer to the centre contour in each frame, this indicates an approaching distractor to the true target. The process of distractor approaching analysis can be seen in Figure 5. On this basis, the approaching distractor can be analysed through the following three steps:

**Step one:** Contour number judgement. If only one contour exists and is located in the central part of the response map, this represents good tracking conditions. Otherwise, if there is more than one contour, distractor approaching analysis is utilised.

**Step two:** Calculate the minimum distance between contours.

(3) $$d_{k,i,j}=\sqrt{{\left(x_{k,i}^{c1}-x_{k,j}^{c2}\right)}^{2}+{\left(y_{k,i}^{c1}-y_{k,j}^{c2}\right)}^{2}}$$

(4) $$d_{k}=\min\left\{d_{k,i,j}\right\}$$

where *k* is the *k*th frame of the tracking sequence; *c*1 represents the contour with the highest response score and *c*2 represents the contour with the second highest response score;$\;i\in \left(0,N_{c1}\right)\;j\in \left(0,N_{c2}\right)$, where $N_{c1}$ and $N_{c2}$ are the total points of contours *c*1 and *c*2; $d_{k,i,j}$ represents the distance between the ith point of contour *c*1 and the jth point of contour *c*2 in the kth frame; and $d_{k}$ is the minimum distance between contours *c*1 and *c*2.

**Step three:** Analyse the trend of the distance change.

(5) $$MD_{k}=\frac{\sum_{k-M\leq n\leq k}\left(d_{n}-d_{n-1}\right)}{M}$$

where *M* means there are *M* frames that are used for analysing the approaching trend, and $MD_{k}$ is the mean different distance in the *M* frames before the kth frame. If $MD_{k}$ is less than a certain threshold $T_{md}$, this indicates that some distractors are approaching.

#### 3.2.3. Object Centre Switching Strategy

Most Siamese trackers choose the location with the highest response score as the target position in each frame, although this strategy can result in tracking drift in certain situations. Consider this situation: if in the (k − 1)th frame, a response peak with a score of 255 (after being normalised) is located in the central part of the response map and another peak with a score of 254 is located at the edge of the response map, while the score of the central peak in the kth frame changes to 254 and the score of the edge peak becomes 255, then the target position will change to the edge peak, ultimately resulting in tracking drift. This is obviously not an ideal tracking strategy when there are distractors nearby.

**Figure 6:**
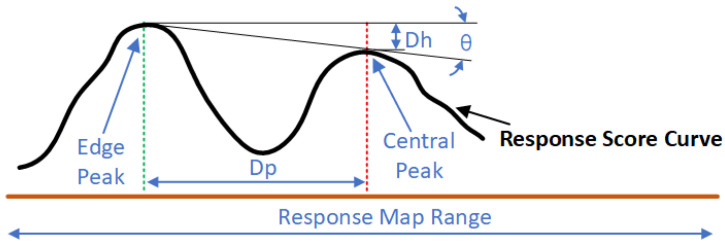
Object centre switching strategy.

In order to address this problem, most existing Siamese trackers employ the strategy of restricting the possible region of the response peak, focusing only on a small region that is close to the position in the previous frame. However, if the peak of the response is at the edge of the response map, it will be abandoned. In certain cases, this strategy can improve performance, but the information of the location of distractors will be lost, and this is useful for further analysis. Unlike traditional Siamese trackers, the proposed method utilises a new strategy that is based on peak angle judgement, as seen in Figure 6.

(6) $$\theta =\arctan\left(\frac{Dh}{Dp}\right)$$

where Dh is the difference in height between two peaks, and Dp is the distance between two peaks. With the proposed strategy, the object centre can only be changed to the edge peak if the angle θ is above a certain threshold.

**Figure 7:**
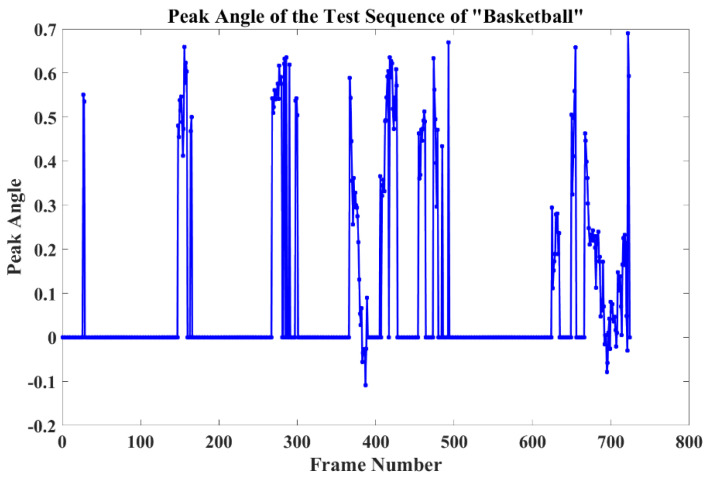
Peak angle plots of sequence ‘Basketball’ of OTB100.

Figure 7 shows that the peak angle θ changes throughout the entire sequence. In these plots, angles larger than 0 demonstrate that there is more than one peak in the response map and the central peak is higher than the edge peak. At the same time, when the angle is less than 0, this means the edge peak will have a higher score, and the rest of the points with an angle equal to 0 indicate only one peak in the response map, which means that no distractors can be found near to the true target. The figure demonstrates that the angle changes from a positive number to a negative number at approximately the 380th and 690th frames, and these frames are distractors that are moving close to the true target.

#### 3.2.4. Pseudo-Code of the Proposed Method

The possibility of distractors approaching and their positions can be calculated by using the above-introduced response behaviour analysis, the pseudo-code can be seen in Algorithm 1.
**Algorithm 1:** Proposed tracking method**Input: **$I={\left\{i_{n}\right\}}_{n=1}^{N}c$ (N is the total number of sequences)
**for** i=1,…N **do**    $R_{i}\leftarrow g_{i}\left(x,z\right)$   # Response map    $PO_{i}\leftarrow \arg\max f\left(x\right)*f\left(z\right)$  # Target position offset
    $NR_{i}\leftarrow \frac{R_{i}}{\max\left(R_{i}\right)}\times 255$   #Normalisation    $C_{i,j}\leftarrow NR_{i}$   # Find j isohypse contours    **if** j>2        $d_{k,i,j}\leftarrow \sqrt{{\left(x_{k,i}^{c1}-x_{k,j}^{c2}\right)}^{2}+{\left(y_{k,i}^{c1}-y_{k,j}^{c2}\right)}^{2}}$   # (Equation (3))        $MD_{k}\leftarrow \frac{\sum_{k-M\leq n\leq k}\left(d_{n}-d_{n-1}\right)}{M}$   # (Equation (5))        **if** $MD_{k}<T_{md}$          $flag_{distracotr}\leftarrow true$      **end if**      $\theta \leftarrow \arctan\left(\frac{Dh}{Dp}\right)$   # (Equation (6))      **if** $\theta >T_{pa}$       $PO_{n}=PO_{i}$      **end if**      **else**       $PO_{n}=PO_{i-1}$   # use the position of previous frame      **end else****else**                $PO_{n}=PO_{i}\;$**end for**

## 4. Experiments and Discussion

The approach in this study was implemented in Python using PyTorch on a PC with Intel i7, 32G RAM, NVIDIA GeForce RTX 3060. In this section, detailed results are provided. All tracking results are provided by official implementations in order to ensure a fair comparison.

### 4.1. Datasets

As a means of verifying the efficiency of the proposed method, it was evaluated using the well-known OTB100, GOT-10k and LaSOT tracking benchmarks. OTB100 [36] consists of 100 videos of 22 object categories with 11 tracking attributes. These attributes include abrupt motion, background clutter, blur and deformation. The average resolution of OTB100 is 356 × 530, while the length ranges between 71 and 3872 frames. GOT-10k [37] consists of 10,000 videos from the semantic hierarchy of WordNet [38]. This is divided into training, validation and test splits. The training split contains 9340 sequences with 480 object categories, while the test split contains 420 videos with 83 object categories, each sequence having an average length of 127 frames. LaSOT [39] is a high-quality benchmark that applies to large-scale single-object tracking. LaSOT consists of 1400 sequences with a total of over 3.5 million frames.

### 4.2. Evaluation Metrics

OTB100 evaluation is based on two metrics: precision plot and success plot.

The precision plot is based on the central location error, which is defined as the average Euclidean distance between the predicted centres of the target object and the ground truth centres in a frame. This is generated by plotting the distance precision over a range of thresholds. Distance precision is defined as the percentage of frames in which the target object is located within a centre location error of 20 pixels.

However, the precision plot does not reflect the size or scale accuracy of the target, so the IoU (Intersection over Union) is utilised for the measurement of prediction error. Given the estimated bounding box p and the ground truth bounding box g, IoU is defined as $\left(p\cap g\right)/\left(p\cup g\right)$. Therefore, the success rate is the percentage of frames in which the IoU is below a certain threshold, and the success plot is generated by varying the overlap threshold from 0 to 1.

For the GOT-10k dataset, the average overlap (AO) and success rate (SR) are utilised as the metrics. AO is measured by calculating the average of overlaps between all ground truth and predicted bounding boxes. SR is measured by calculating the percentage of successfully tracked frames where overlaps exceed a certain threshold. In the evaluation, AO is exploited for the overall performance ranking.

### 4.3. Implementation Details

**Training:** The parameters that were used in the training stage were the same as SiamFC. The ILSVRC15 dataset was used, and the training was performed over 50 epochs, each consisting of 50,000 sampled pairs. The gradients for each iteration were estimated using mini-batches of size 8, and the learning rate was annealed geometrically at each epoch from 10^−2^ to 10_−5_.

**Tracking:** Unlike with classic SiamFC, the proposed response analysis was added to the tracking pipeline as a means of optimising tracking accuracy.

**Figure 8:**
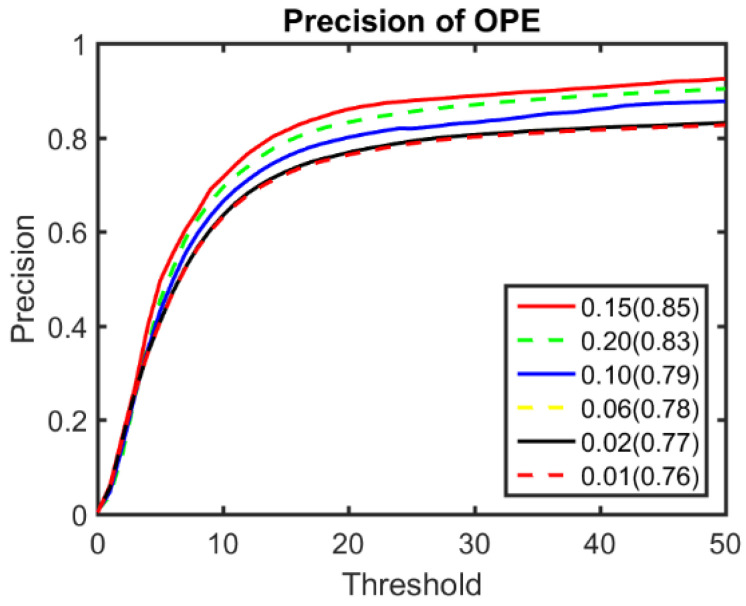
Experimental results of different angle thresholds of the OTB100 benchmark.

The peak angle threshold $T_{pa}$ that was used in the peak switching strategy was determined through experiments (as can be seen in Figure 8) and was set to 0.15. The $T_{md}$ in distractor approaching analysis was set to 15, and the frame number M was set to 10.

### 4.4. Performance Evaluation

For evaluating the performance of the proposed method, which is known as SiamFC-RBA, the tracker was compared against six different trackers: SiamFC, SiamRPN, DaSiamRPN, CFNet, CSK and Staple. SiamFC is the classic Siamese tracker, and SiamRPN is an advanced Siamese tracker that exhibits state-of-the-art performance.

In addition, the response behaviour analysis module was embedded into DiMP, named DiMP-RBA, as a means of testing the effectiveness of the proposed response behaviour analysis module in the majority of response map-based trackers.

**Figure 9:**
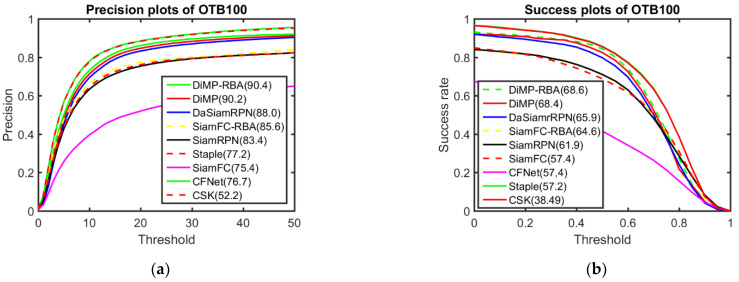
Comparison between the proposed method and baseline trackers on the OTB100 benchmark: (**a**) precision plots; (**b**) success plots.

The precision and success plots of OTB100 can be seen in Figure 9. The results demonstrate that the two DiMP-based trackers performed better than the others, and the DiMP-RBA that uses the proposed response behaviour analysis method was approximately 0.2% more precise than the original DiMP. The precision of SiamFC-RBA was 10% greater than that of the classic SiamFC and approximately 0.2% higher than that of SiamRPN, but it was 0.3% lower than the state-of-the-art tracker DaSiamRPN. Although the result of the tracker in this study is almost at the same level as DaSiamRPN, as the training structure was not rebuilt, the same training result as classic SiamFC was used and the update strategy was modified during the tracking process, the performance is still quite impressive. The same phenomenon can be observed in the success plot result. It can also be seen that the proposed method, DaSiamRPN and SiamRPN performed far better than the four other trackers (SiamFC, CFNet, CSK and Staple).

**Figure 10:**
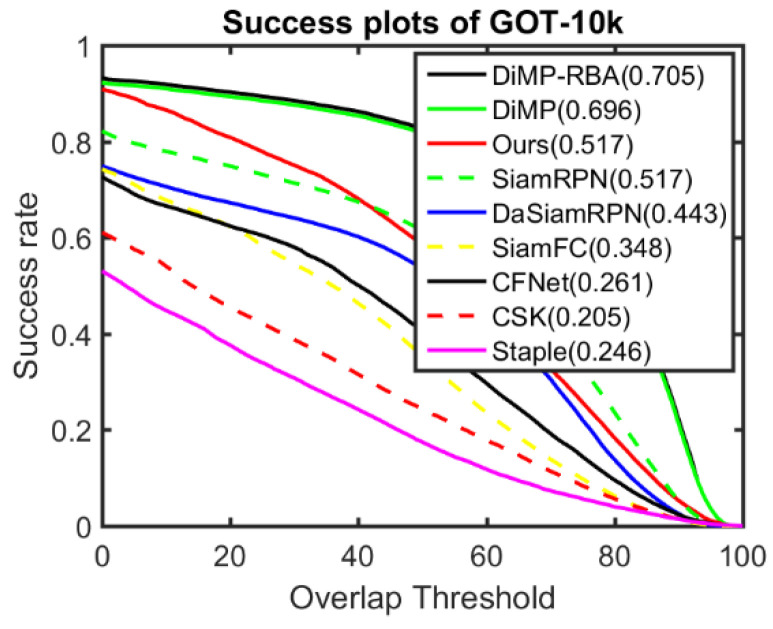
Comparison between the proposed method and baseline trackers on the GOT-10k benchmark.

The comparison results for GOT-10k are shown in Figure 10. The performances of the two DiMP-based trackers were far better than those of the other trackers, and the DiMP-RBA which utilises the proposed response behaviour analysis method had approximately 0.9% better precision than the original DiMP. The overall scores of the tracker in this study and SiamRPN are almost identical (0.517) and far better than those of the four other trackers. Although the proposed tracker and SiamRPN have similar scores, they exhibit different performance patterns. When the overlap threshold was below 40, the tracker in this study exhibited better performance than SiamRPN, whereas SiamRPN was better in the opposite situation.

**Figure 11:**
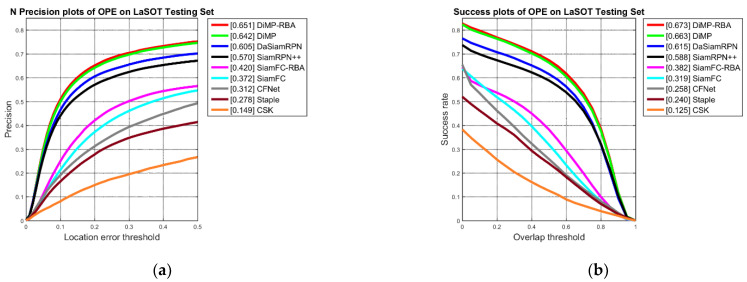
Comparison between the proposed method and baseline trackers on the LaSOT benchmark: (**a**) precision plots; (**b**) success plots.

The comparison results of LaSOT can be seen in Figure 11. The overall trend is the same as for the other two benchmarks. The performance of DiMP-RBA was improved by approximately 1% following the use of response behaviour analysis, and SiamFC-RBA demonstrated both a higher precision and success rate than the original SiamFC and the remaining trackers.

**Figure 12:**
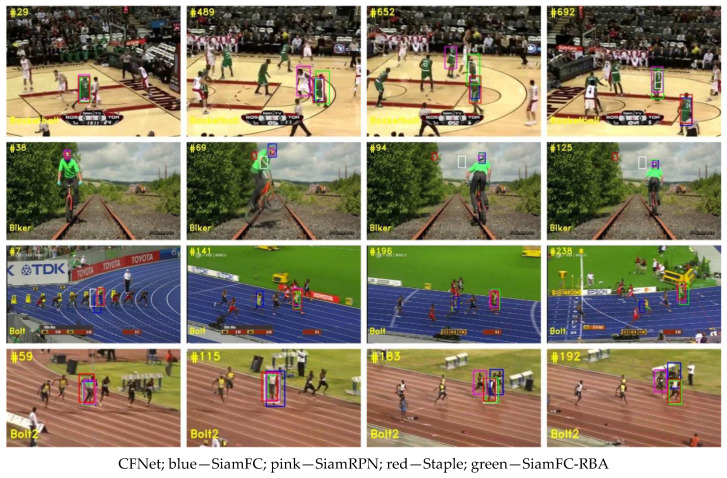
Qualitative results for some typical challenging scenarios.

The experiment results are shown in Table 1, and the qualitative results for some typical challenging scenarios are shown in Figure 12. The performance of the proposed method was the same as that of DaSiamRPN. The tracker in this study was better with the GOT-10k benchmark than DaSiamRPN, while the opposite is true with the OTB100 benchmark. As has previously been mentioned, as the training structure was not rebuilt, meaning that the same training result as the classic SiamFC was used and the update strategy was just modified during the tracking process, the performance of the proposed method showed a different method for addressing the problem of tracking drift in background clutter scenarios. This proves that this type of strategy can also be used to achieve the state-of-the-art level.

**Figure 13:**
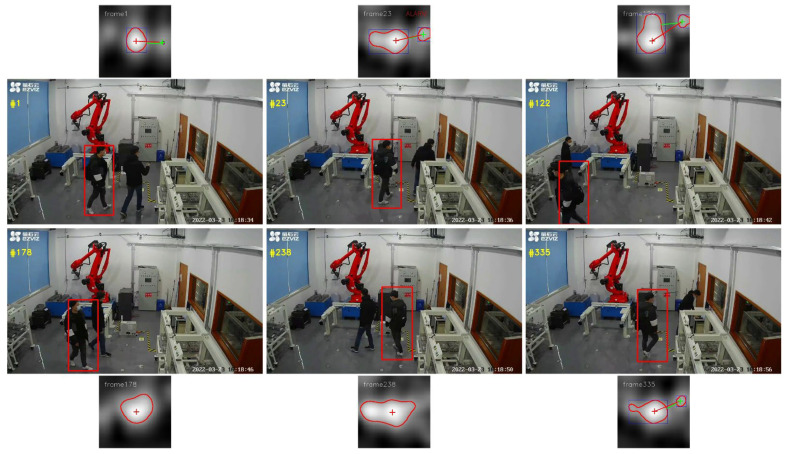
Human tracking with similar objects online in real time.

In addition to the benchmark dataset evaluation, the algorithm was implemented using the online real-time video stream of the surveillance camera that is installed in our laboratory. In this scenario, two men wearing similar clothes walked into the lab and crossed paths several times. The distractor approaching process can be seen in the corresponding response map in Figure 13, with the tracker still performing well in most cases. Due to the data of the new scenario not being included in the benchmark and the situation with real-time online application, the performance of the method that was used in this study was not compared to other trackers.

## 5. Conclusions

This paper proposes an improved SiamFC tracker that is based on response map analysis as a means of addressing the problem of tracking drift in background clutter scenarios. The key point of this method is that it can be used for judging whether there are distractors near the real target by analysing the behaviour of the response map and by updating the target positioning strategy on the basis of this information. Extensive experiments on visual tracking benchmarks including OTB100, GOT-10k and LaSOT found that by using the proposed method, in comparison to the original SiamFC, the precision performance of SiamFC-RBA increased by approximately 8%, 16% and 5%, respectively, while also outperforming SiamRPN, CSK, CFNet and Staple. The response behaviour analysis module was also embedded into DiMP, which is known as DiMP-RBA, for testing the effectiveness of the proposed response behaviour analysis module in most response map-based trackers. The experimental results found that DiMP-RBA outperformed the original DiMP by 0.2%, 0.9% and 0.9%, respectively, in the three benchmarks. Although the DiMP improvement was relatively small, this proved that the proposed response behaviour analysis module can be embedded into other response map- or score map-based trackers as a means of improving tracking performance.

## Figures and Tables

**Table 1:** Comparison between the proposed method and baseline trackers on the OTB100, GOT-10k and LaSOT benchmarks. Red and blue indicate the two trackers that use the proposed response behaviour analysis method.

Trackers	OTB100		GOT-10k		LaSOT		FPS
	Precision	Success	AO	SR	Precision	Success	
DiMP -BRA	0.904	68.6	0.705	0.819	0.651	0.673	15.1
DiMP	0.902	68.4	0.696	0.816	0.642	0.663	15.2
DaSiamRPN	0.88	65.9	0.444	0.53	0.605	0.615	134.4
SiamFC-RBA	0.85	62.6	0.517	0.584	0.420	0.382	42.7
SiamRPN	0.83	61.9	0.517	0.615	0.570	0.588	3.17
SiamFC	0.77	57.4	0.348	0.353	0.372	0.319	43.8
CFNet	0.76	57.4	0.261	0.243	0.312	0.258	2541
Staple	0.77	38.49	0.246	0.248	0.278	0.240	28.7
CSK	0.52	57.2	0.205	0.174	0.149	0.125	133.3

## Data Availability

Not applicable.

## Source Code

The code for this paper is available at: https://github.com/cramkl/SiamFC-RBA, accessed on 27 August 2022.

## Abbreviations

Symbols and abbreviations	Full meaning
$p_{t}$	Target template patch
$p_{s}$	Search patch
ρ	Learnable parameter of Siamese trackers
b	Scalar offset value
q	Central position of the target
$g_{\rho }\left(p_{t},p_{s}\right)$	Response map denoting the similarity between $p_{t}$$\;\mathrm{and}\;p_{s}$
$x_{k,i}^{c1}$	x coordinate of the ith point in the *c*1 contour of the kth frame
$d_{k,i,j}$	Distance between the ith point of contour *c*1 and the jth point of contour *c*2 in the kth frame
$MD_{k}$	Mean distance before the kth frame
$R_{i}$	Response map of the ith frame
$NR_{i}$	Normalised response map of the ith frame
$C_{i,j}$	The jth contour of the ith frame
$T_{md}$	Distance threshold
θ	Angle between the two highest peaks
AO	Average overlap
SR	Success rate
IoU	Interaction over Union
FPS	Frame per second
